# Authors’ Response to Peer Reviews of “Impact of a Point-of-Care Ultrasound Training Program on the Management of Patients With Acute Respiratory or Circulatory Failure by In-Training Emergency Department Residents (IMPULSE): Before-and-After Implementation Study”

**DOI:** 10.2196/72092

**Published:** 2025-03-03

**Authors:** Sandra Bieler, Stephan von Düring, Damien Tagan, Olivier Grosgurin, Thierry Fumeaux

**Affiliations:** 1 Médecin cheffe, Service des Urgences, Hôpital de Sion, Sion, 1950, Switzerland; 2 Faculté de Médecine de l’Université de Genève, Hôpitaux Universitaires de Genève, Genève, Switzerland; 3 Service des Soins critiques, Hôpital Riviera Chablais, Rennaz, Switzerland; 4 Service de médecine interne générale et Service des Urgences, Hôpitaux Universitaires de Genève, Genève, Switzerland; 5 Hirslanden Geneva Clinics, Geneva, Switzerland

**Keywords:** point-of-care ultrasonography, training program, acute respiratory failure, acute circulatory failure, emergency department

*This is the authors’ response to peer-review reports for “Impact of a Point-of-Care Ultrasound Training Program on the Management of Patients With Acute Respiratory or Circulatory Failure by In-Training Emergency Department Residents (IMPULSE): Before-and-After Implementation Study.”*

## Round 1 Review

### Anonymous [1]

#### General Comments

*This paper [2] researches an essential component of point-of care ultrasonography. As this modality is rapidly evolving, evaluation of the impact on patient management and outcomes as well as cost-effectiveness is essential. Both subjects discussed in the paper result in a highly relevant manuscript. Even though the authors discuss relevant subjects, there are some problems with the manuscript.*

#### Specific Comments

##### Major Comments

*1. The title of the manuscript suggests that the authors researched the impact of ultrasound after implementation. However, as stated in the Methods section, ultrasound is already used by senior physicians. Thus, the impact of ultrasound after implementation is not researched but rather the impact of ultrasound used by residents. I suggest that the authors clarify that this is a feasibility and impact study on the implementation of point-of-care ultrasound (POCUS) used by residents in the emergency department (ED) in the title and Abstract.*

Response: The title has been modified according to the reviewers’ indications, to highlight the fact that the study’s primary aim is to validate the implementation of a training curriculum for interns in training, and not to study the effect on patient outcome.

*2. The authors state that patients were not included consecutively due to logistics in phase 2. This results in a high risk of bias in the included patients. Please include in the CONSORT (Consolidated Standards of Reporting Trials) diagram the number of patients that were eligible and were excluded based on exclusion criteria or missed.*

Response: As mentioned, the patients were not fully consecutively included due to organizational reasons: an incoming patient could only be considered for inclusion if the emergency department (ED) patient flow allowed, without delaying treatment or impacting on department operations. This is mentioned in the text. However, the number of patients who could have been included is not known (no traceability of screening).

*3. It is unclear how many residents were performing the ultrasound examinations included in the analysis: the Methods section state that there was only 1 resident at the ED in both phases, while in the Results section, it states that there were 12 residents trained. Please clarify.*

Response: Twelve doctors were trained, but only 1 resident at a time worked in the ED during each shift, and only he or she could therefore include patients during that shift, as specified in the text. We hope that the text will clarify this point.

*4. The authors state that they chose a before-and-after implementation to evaluate the effect of POCUS to avoid contamination. However, before the implementation, POCUS was already used by senior physicians, which raises the question if POCUS was indeed not used in phase 1 of the trial.*

*5. Interestingly, in the Discussion section, the author discussed that the publication of Msolli et al did not find an improvement of diagnostic accuracy. It would be interesting to discuss why this is the case.*

Response: As suggested by the reviewer, we have added a comment on the difference in the diagnostic accuracy of point-of-care ultrasound (POCUS) in our study and in the study by Msolli et al [3].

*6. In the Discussion and Conclusion, it is suggested that the use of POCUS might lead to a decrease in hospital mortality. Since this is an observational study in which, just as the authors state, a diagnostic tool rather than a therapeutic intervention is researched, this is too rash to state. Please remove this from the Conclusion and Abstract.*

Response: We have modified the Conclusion to relativize the effect of implementation on mortality, which is at best indirect, as mentioned by the reviewer.

##### Minor Comments

###### Overall

*7. The authors provide results with IQR; however, no ranges are given. Please describe results as mean (SD) when data are normally distributed or median (25th percentile – 75th percentile) when data are not normally distributed.*

Response: As all data are not normally distributed, we have chosen to keep the IQR (25th-75th), so as not to overload the text.

*8. Formatting of the full manuscript needs some attention. For example, in the Abstract, not all sentences start with a capital letter. Also, it is common in the English language to write number in full up to 20.*

*9. Please follow the author guidelines of the journal for reporting values and the structure of the manuscript.*

Response: Formatting has been adapted according to the transmitted comments.

###### Title Page

*10. The authors state that a clinical trial registration was done. However, it seems that they refer to a registration by a medical ethical review board. Please provide a clinical trial registration or if not applicable, remove it from the title page.*

Response: We have deleted the information on registration.

###### Introduction

*11. In the first sentence, please state the full name of “emergency department” before using the abbreviation ED.*

###### Methods

*12. Figure 1 should be formatted. The most common formatting is according to the CONSORT flow diagram.*

Response: We have formatted Figure 1 according to the instructions.

###### Results

*13. Please do not discuss the results in the Results section.*

Response: We have deleted all discussions of the results in the Results section.

###### Discussion

*14. Please end the Discussion section with the strengths and limitations. The secondary findings should be above the Strengths and Limitations section.*

Response: We have moved the secondary findings to before the discussion on the strengths and limitations.

## Round 2 Review

### Anonymous

*I would like to compliment the authors of their extensive changes to the manuscript. I have some minor comments.*

Response: We thank the editor and the reviewer for their careful reading of our manuscript and for their valuable comments. We have addressed all issues raised by them and modified the text accordingly. We have uploaded a change tracking version of the manuscript, with changes highlighted in yellow.

Before-and-after design: In such a study design, the only difference between the two phases should be the implemented intervention. In IMPULSE (Impact of a Point-of-Care Ultrasound Examination), the intervention was the implementation of immediate POCUS examination by junior in-training residents managing patients in the first line, after a short structured training program. This was performed only during the postimplementation phase, and never done before. POCUS could be performed in both phases by senior experienced physicians, but later in the management of the patient, after the initial clinical evaluation (and after the POCUS during the postimplementation phase) of the junior resident. We therefore continue to affirm that this is indeed a before-and-after study design, with a clear implementation of a changing practice. We have clarified this in all sections of the text.

We have, as suggested, included information on the residents’ characteristics, as this valuable information is important for the interpretation of the study results. A new section has been added in the Methods and in the Results parts of the text.

We have put the 25th‐75th IQR range everywhere in the text and tables, as suggested.

We have removed the figure legends from the uploaded figures.

As mentioned, a change-tracking version has been uploaded as a supplementary file, with changes highlighted in yellow.

All ethics information has been grouped in a specific section in the Methods part of the text.

We have followed the guidelines on reporting results.

#### Minor Comments

*1. I would suggest changing the sentence “However, there is still no strong evidence that the diagnostic accuracy of POCUS translates into a clinically relevant difference in patient outcomes” in the Introduction, because you also do not provide strong evidence (I do not know if we ever could provide strong evidence). I would suggest that you focus it more on the fact that the impact of using POCUS in the management of patients in the ED is still relatively unknown.*

Response: We have adapted the sentence on the evidence of the clinical impact of POCUS in the Introduction, as suggested by the reviewer.

*2. I would suggest to start the Discussion section with a short summary of the key findings.*

Response: We have started the Discussion section with a short summary of key findings.

## Abbreviations

ED: emergency department
IMPULSE: Impact of a Point-of-Care Ultrasound Examination
POCUS: point-of-care ultrasound
